# mRNA-Based Anti-TCR CDR3 Tumour Vaccine for T-Cell Lymphoma

**DOI:** 10.3390/pharmaceutics13071040

**Published:** 2021-07-07

**Authors:** Marina Tusup, Severin Läuchli, Natalia Teresa Jarzebska, Lars E. French, Yun-Tsan Chang, Maya Vonow-Eisenring, Andreas Su, Thomas M. Kündig, Emmanuella Guenova, Steve Pascolo

**Affiliations:** 1 Department of Dermatology, University Hospital of Zürich, Raemistrasse 100, 8091 Zürich, Switzerland; marina.tusup@usz.ch (M.T.); severin.laeuchli@usz.ch (S.L.); NataliaTeresa.Jarzebska@usz.ch (N.T.J.); thomas.kuendig@usz.ch (T.M.K.); 2 Faculty of Medicine, University of Zürich, 8006 Zürich, Switzerland; 3 Faculty of Science, University of Zürich, 8006 Zürich, Switzerland; 4 Department of Dermatology and Allergy, University Hospital, LMU Munich, 80539 Munich, Germany; lars.french@med.uni-muenchen.de; 5 Dr. Phillip Frost Department of Dermatology & Cutaneous Surgery, Miller School of Medicine, University of Miami, Miami, FL 33146, USA; 6 Department of Dermatology, Lausanne University Hospital (CHUV), University of Lausanne, 1000 Lausanne, Switzerland; yun-tsan.chang@chuv.ch; 7 Department of Immunology, University Hospital of Zürich, Raemistrasse 100, 8091 Zürich, Switzerland; Maya.Vonow-Eisenring@usz.ch; 8 BioNTech, 55131, Mainz, Germany; Andreas.Su@biontech.de

**Keywords:** in vitro transcribed mRNA, ivt mRNA, vaccine, T-cell lymphoma, CTCL, TCR, CDR3

## Abstract

Efficient vaccination can be achieved by injections of in vitro transcribed mRNA (ivt mRNA) coding for antigens. This vaccine format is particularly versatile and allows the production of individualised vaccines conferring, T-cell immunity against specific cancer mutations. The CDR3 hypervariable regions of immune receptors (T-cell receptor, TCR or B-cell receptor, BCR) in the context of T- or B-cell leukaemia or lymphoma are targetable and specific sequences, similar to cancer mutations. We evaluated the functionality of an mRNA-based vaccine designed to trigger immunity against TCR CDR3 regions in an EL4 T-lymphoma cell line-derived murine in vivo model. Vaccination against the hypervariable TCR regions proved to be a feasible approach and allowed for protection against T-lymphoma, even though immune escape in terms of TCR downregulation paralleled the therapeutic effect. However, analysis of human cutaneous T-cell lymphoma samples indicated that, as is the case in B-lymphomas, the clonotypic receptor may be a driver mutation and is not downregulated upon treatment. Thus, vaccination against TCR CDR3 regions using customised ivt mRNA is a promising immunotherapy method to be explored for the treatment of patients with T-cell lymphomas.

## 1. Introduction

Vaccines based on in vitro-transcribed messenger RNA (ivt mRNA) have been shown to trigger specific immunity against the encoded antigen (cancer-associated antigens or proteins from pathogens) [1,2,3,4,5,6]. Several formulations are currently being evaluated in clinical studies for the treatment of cancers (e.g., melanoma, lung carcinoma) and infectious diseases (e.g., SARS-CoV-2, rabies, Zika virus, influenza) [7,8,9,10]. During the COVID-19 pandemic, the mRNA vaccines were the first to be tested in humans as a prophylaxis against SARS-CoV-2 infection and the first to be approved [11]. Their implementation on a global scale is helping to curtail the current pandemic. In the context of anticancer vaccination, three hallmark features of ivt mRNA are essential for the success of the approach [12,13,14,15]: (i) the ease of production [16] allows an individualised vaccine design whereby each patient is for example immunised using a customised ivt mRNA coding for individual tumour mutations, (ii) the TLR agonistic feature of nonmodified RNA triggers innate immunity that can result in expression of type I interferon and (iii) efficient transfection of antigen presenting cells by naked or formulated ivt mRNA allows efficient triggering of a T-cell response against the encoded protein. In addition, as the nonreplicating ivt mRNA vaccine is transient and unable to integrate into the genome, this approach is believed to be a safe form of gene therapy. Vaccinating cancer patients against the mutanome involves the sequencing of the tumour’s genome, a comparison to the healthy tissue, and in silica prediction of relevant coding mutations capable of providing MHC-presented peptides before the design and production of the individualised mRNA vaccine through a process named MERIT (mutanome engineered RNA immunotherapy) [17,18]. Many mutations can be evidenced by this method and they can be targeted by vaccination. Ideally, those mutations are located in highly expressed genes (for more details on the identity of mutations that can be targeted and their selection for individualised vaccines, see the review by Vormehr et al. [19] and the original article by Kreiter et al. [13]). One faster way to identify tumour-specific mutated protein sequences (i.e., mutated MHC epitopes) can be found for leukaemic or lymphoblastic cells by focusing on the hypervariable loops of the immune receptors (TCR or BCR). These loops are not encoded by the wild-type genome as they result from the random joining of segments and eventual trimming or addition of bases; therefore, they can be considered mutation-like sequences specific to a tumour T- or B-cell. Indeed, the clonotypic receptor for B-cell leukaemia has been recently proven to be a driver mutation for the disease [20]. Therefore, leukaemic cells cannot escape an immune response directed against their specific BCR. Indeed, vaccinations against BCR using, for example, DNA vaccines were shown to provide some protection against murine B cell lymphoma [21]. Here, we evaluated for the first time the possibility of using a designed mRNA vaccine to immunise against the TCR hypervariable CDR3 regions for the treatment of a mouse syngeneic T-cell lymphoma model (the EL4 T cell lymphoma cell line in C57BL/6). We demonstrated that this vaccination strategy can provide some protection against T-cell tumours.

## 2. Materials and Methods

### 2.1. Production of Ivt mRNA

The academic ivt mRNA production and formulation platform in Zurich (http://www.cancer.uzh.ch/en/Research/mRNA-Platform.html, accessed on 21 May 2021) provided mRNA coding for firefly luciferase and EL4-TCR-CDR3s [22]. The mRNA was not modified: it contained the four canonical nucleotides and was therefore immunostimulatory [23,24]. The 5′ end consisted of a CleanCap ^TM^ Reagent AG (TriLink N-7113, San Diego, CA, USA) (co-transcriptional capping) and an eIF4G-binding aptamer [25]. The 3′ end consisted of an optimised stabilisation sequence [26] and a poly-A tail.

### 2.2. Cells and FACS

The EL4 mouse lymphoma T-cells were available in our Department of Dermatology at the University Hospital of Zürich, Zürich, Switzerland and maintained in a complete medium containing: RPMI medium (Thermo Fisher Scientific, Waltham, MA, USA), 10% foetal calf serum (FCS), Glutamine 5% and 0.2% of the antimicrobial reagent Normocin (InvivoGen, San Diego, CA, USA). For the analysis of surface receptors, cultured cells (originating from tumours dissected from mice on day 10 and digested with 0.05% Collagenase IV (Sigma-Aldrich, St. Louis, MO, USA) at 37 degrees before being homogenised and filtered through 70 µm meshes) were resuspended at 10 millions per ml in 100 microlitres of complete medium with a final concentration of 1 μg/mL of fluorescent antibodies: PE TCR V beta 12 and FITC TCR alpha beta (Invitrogen, Waltham, MA, USA).

### 2.3. Patient and T Cell Receptor (TCR) Clonality Assessment by Flow Cytometry

Peripheral blood and skin from the patients with CTCL were collected in the context of the University of Zürich Biobank, funded by the University of Zurich University Research Priority Program (URPP) in translational cancer biology. All patients signed an informed consent agreeing to the use of samples, including the generation of cell cultures according to the Biobank project (EK No. 647, approved on the 25 October 2017). The study was conducted in accordance with the principles of the Declaration of Helsinki and was approved by the Institutional Review Board of the University of Zurich (KEK-ZH-Nr. 2015–0209). Vβ clonal T-cell populations were assessed using IOTest ® Beta Mark TCR V beta Repertoire Kit. (Beckman Coulter, # IM3497, Chaska, MN, USA) according to the manufacturer’s instructions.

### 2.4. Animals and In Vivo Experiments

Our studies “Anticancer therapies based on RNA” and “Testing and optimising anti-cutaneous T-cell lymphoma immunological treatments in mice” were approved respectively on 18 January 2018 and 16 December 2020 by the Veterinary Office and its research ethics review committee of the University of Zurich (Kanton Zürich, Health Direction, Veterinary Office, Zollstrasse 20; 8090 Zurich; license number ZH215/17). Animals (as no gender bias is expected in this study, we used only female mice) were purchased from Envigo (Indianapolis, IN, USA). Four- to eight-week-old C57BL/6 mice were injected intravenously with 20 micrograms of mRNA formulated with spleen-specific liposomes from BioNTech “(as previously reported by Kranz et al.) [12]. This liposome is not commercially available, it is in clinical development. It was provided by BioNTech and was used as specified by the company. Three hours post injection, blood was drawn to prepare serum that was used to quantify interferon-alpha by ELISAs (PBL mIFN alpha ELISA Kit, Piscataway, NJ, USA). One week after a boost vaccine, mice subcutaneously received 1 million EL4 cells in 100 microlitres of PBS. Starting at day 5 post tumour injection, the tumour size was evaluated using a calliper. Tumour volume was calculated according to the formula: longest dimension × shortest dimension × shortest dimension/2. Our endpoint was average tumour size until the death of the first mouse. No chemotherapeutic treatment was used as a positive control. For in vivo imaging experiments, 10 µg of synthetic mRNA coding firefly luciferase was formulated in the spleen-specific liposome from BioNTech and injected intravenously. Three hours after mRNA injection, in vivo bioluminescence imaging was performed on an IVIS Lumina instrument (PerkinElmer, Waltham, MA, USA). Before each measurement, d-luciferin (Synchem, Felsberg, Germany) dissolved in PBS (15 mg/mL stock) and sterile filtered was injected (150 μg/g intraperitoneally). Emitted photons from live animals were quantified 10 min post luciferin injections, with an exposure time of 3 min.

## 3. Results

### 3.1. Design of an Ivt mRNA Coding for the TCR CDR3 Regions

**Figure 1:**
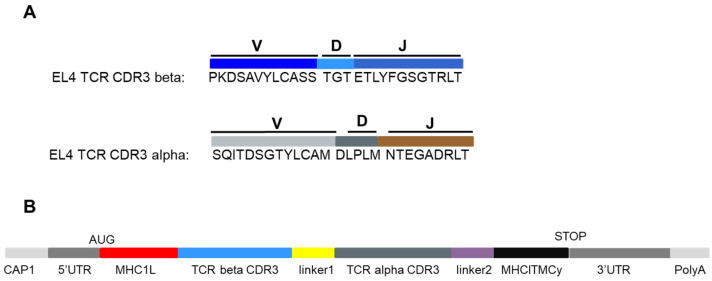
Design of the mRNA vaccine. The V(D)J sequence of the mouse EL4 TCR alpha and beta chains is given in (**A**). In (**B**), the structure of the ivt mRNA is provided. UTR: untranslated region, AUG: start codon, MHC1L: leader sequence of an MHC class I molecule; TCR beta CDR3: the VDJ sequence; linker1: GSAGSAAGSG; TCR alpha CDR3: the VJ sequence; Linker2: GGSGGGGSGG; MHC1TMCy: transmembrane and cytoplasmic domains for H-2 D; STOP: UAG codon and PolyA: poly-A tail.

**Figure A1:**
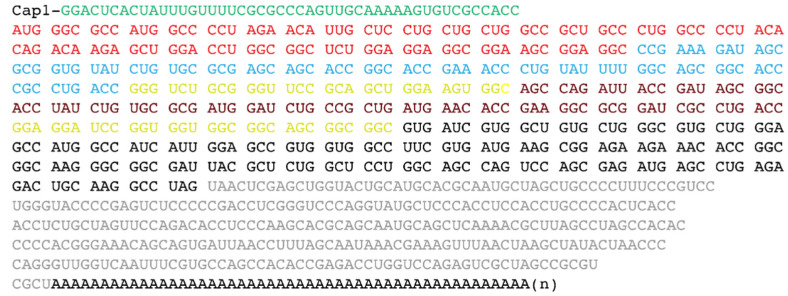
Sequence of the mRNA vaccine. The sequence of the mRNA vaccine is depicted with each functional domain highlighted in colour: in green the 5′ UTR (eIF4G aptamer), in red the leader sequence, in blue the TCR beta CDR3, in yellow the linkers, in brown the TCR alpha CDR3, in black the transmembrane and cytoplasmic domains and in grey the 3′ UTR.

The sequence of the mouse EL4 TCR was published [27], and we delineated the 27 amino acids corresponding to the beta and alpha CDR3s (Figure 1A). We designed a vaccine mRNA incorporating all known optimal sequences (Figure 1B): cap 1 at the 5′ end, a 5′ untranslated sequence corresponding to an eIF4G aptamer, a start codon in the Kozak sequence followed by an MHC class I leader sequence that directs the nascent polypeptide into the endoplasmic reticulum lumen. The sequence encoding the 27 amino acids of the EL4 TCR beta chain follows. Thereafter, a sequence coding for a ten amino acid linker (GSAGSAAGSG) was introduced before the sequence coding for the EL4 TCR alpha chain. Then, a second linker (GGSGGGGSGG) precedes the MHC class I transmembrane and cytoplasmic sequences. The GS-rich linkers commonly used [28]. Besides the usual GGSGGGGSGG linker we used the GSAGSAAGSG linker (a shorter version of the linker described by Chen et al. [29]) which provides more flexibility on the DNA level to prevent homologous recombination between sequences coding for the two linkers. A stop codon ended the open reading frame, and an optimised stabilising 3′ UTR [26] was incorporated before the poly-A tail. The leader, transmembrane and cytoplasmic MHC segments bring the polypeptide to endosomes, favouring the loading of MHC molecules [30]. The complete nucleotide sequence of the synthetic mRNA construct is shown in Appendix A (Figure A1).

### 3.2. Administration of CDR3-Encoding mRNAs Induces Resistance towards EL4

**Figure 2:**
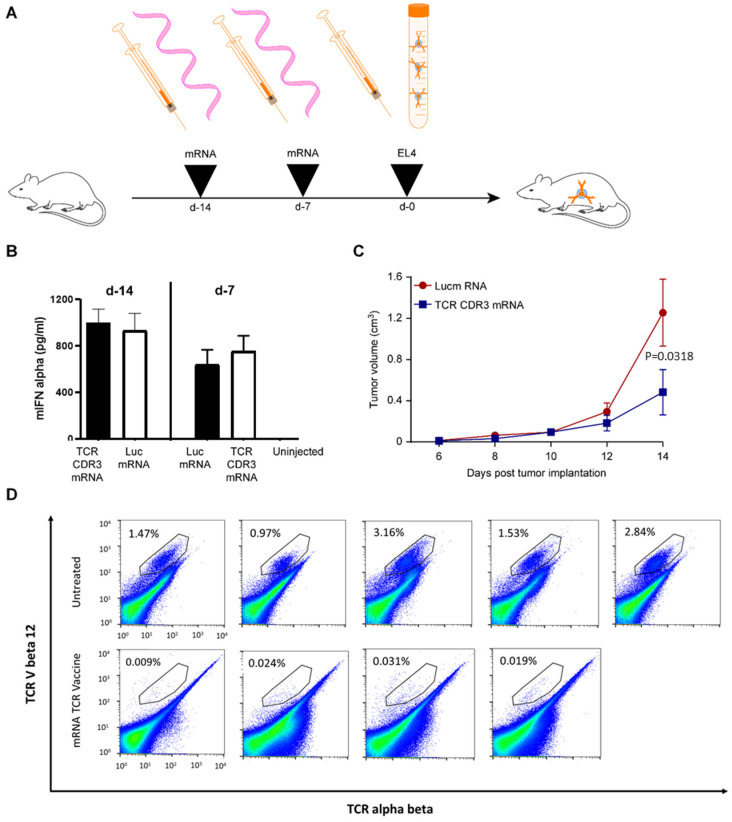
Efficacy of the anti-TCR CDR3 mRNA vaccine in vivo. The synthetic mRNA depicted in Figure 1 was formulated in liposomes and injected intravenously according to the schedule presented in (**A**). Three hours after injection, serum was analysed for interferon-alpha content (five C57BL/6 mice per group). The results are presented in (**B**) for both the priming (d-14) and boost (d-7) vaccines. One week after the boost vaccine, mice (five C57BL/6 mice per group) were implanted subcutaneously with EL4 cells, and the growth of the tumour was measured using a calliper (**C**). A significant (2way ANOVA *p*-value 0.0318) decrease in tumour growth was observed in the mice vaccinated by the mRNA encoding the CDR3 regions of the EL4 TCR. In a follow-up experiment (Figure A2), tumours were collected. Cells from these tissues were grown for one week in vitro, stained using an anti-mouse TCR antibody and an anti-mouse TCR Vbeta 12 antibody and analysed by FACS (**D**). The percentages of TCR V beta 12 and TCR alpha beta double-positive cells (gate is shown on the dot plots) in the cultures are indicated.

**Figure A2:**
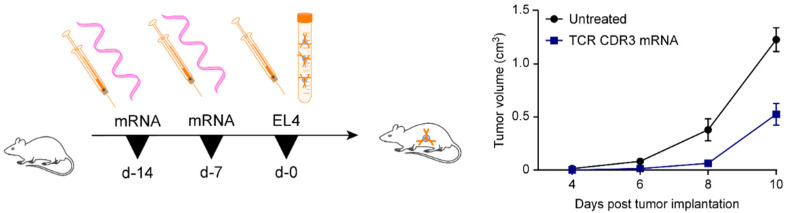
Efficacy of the anti-TCR CDR3 mRNA vaccine in vivo. The experiment presented in Figure 2 was repeated and gave a similar result: mice vaccinated with the mRNA (five C57BL/6 mice) encoding the EL4 TCR CDR3s had a significant (2way ANOVA p value 0.0008) delay in tumour growth compared to untreated (six C57BL/6 mice).

For vaccination, ivt mRNA was formulated in a liposome that is injected intravenously and delivers nucleic acids to antigen presenting cells in secondary lymphoid organs as presented by Kranz et al. [12]. The vaccination scheme consisted of two injections separated by one week before tumour challenge (Figure 2A). In order to assess the effectiveness of the intravenous injections and the functionality of the formulations in delivering RNA to the immune cells, we measured interferon-alpha in the serum of the animals three hours after injection (Figure 2B). As expected [12], both ivt mRNAs (coding for the EL4 CDR3s or for a negative control luciferase) induced strong type I interferon production upon priming and boosting with ivt RNA. Subsequently, subcutaneous inoculation with the EL4 T cell lymphoma cell line resulted in clearly detectable tumour growth in all mice, but a significant delay (2way ANOVA, *p* value 0.0318) was observed in the group of mice that received the mRNA coding for the EL4 CDR3 regions (Figure 2C). Testing in vitro the interferon-gamma production by splenocytes from those mice either cocultured with EL4 cells or transfected with the CDR3-coding mRNA, we could not record a significant difference between vaccinated and control group (data not shown). This result is probably due to the low frequency of EL4- or TCR-specific T-cells in the spleen. Thus, to assess the potential immunological impact of the vaccination on the tumour cells, in a repetition of the experiment (Figure A2), the tumours were collected at day 10 and dissociated, and the cells were grown for one week. Staining of the cell mixture revealed expression of the specific for EL4 TCR V beta 12 in a fraction of the cells obtained from tumours of nonvaccinated mice, while no cell culture made from the tumours that grew in the TCR CDR3 mRNA-vaccinated mice had such frequencies of TCR V beta 12-positive cells. This result indicates that in the TCR CDR3 vaccinated mice, the triggered immune response induced a selective growth of EL4 cells that had lost the TCR expression. This finding demonstrates that in EL4 cells, which are highly proliferative, TCR expression is not required for growth in vitro and in vivo. Therefore, immune escape variants can appear. The selection of V beta 12-negative EL4 cells in all TCR CDR3 mRNA-vaccinated mice and in none of the control mice can be only explained by immunoediting [31] meaning that an adaptive anti-TCR immunity has been triggered by the prophylactic vaccine. Since our in vitro studies failed to evidence a T-cell response, it cannot be excluded that several arms of immunity (cytotoxic T-cells, helper T-cells or B-cells) have been induced by the TCR CDR3 mRNA vaccine and participate in the selection of V beta 12-negative EL4 cells.

### 3.3. Retention of the TCR by CTCL

**Figure 3:**
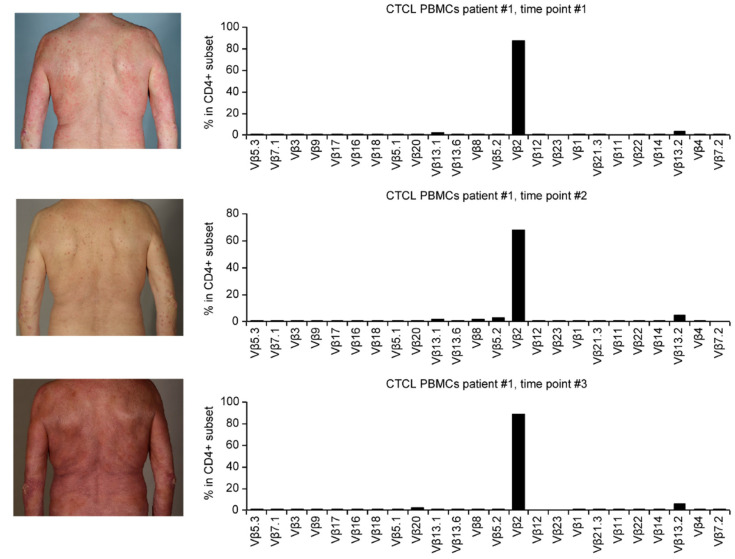
Time course and continuous detection of the same TCR Vbeta2 during the course of the disease. In blood cells from a CTCL patient over one year, the tumour cells express the TCR at the cell surface (FACS analysis), and no downregulation of this molecule was observed. There are six months between the time points. Between time point #1 and time point #2 the patient received immunomodulatory treatment by interferon-alpha that resulted in complete regression of skin lesions. Between time point #2 and time point #3 the patient received no therapy (“watchful waiting”) and relapsed.

**Figure A3:**
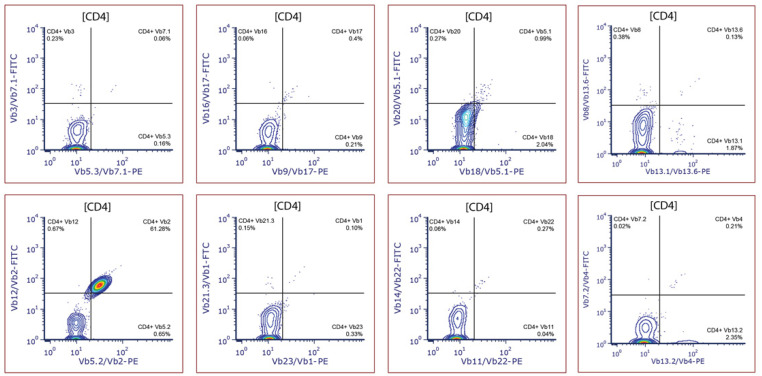
Example of TCR Vbeta clonal detection (Vbeta 2) in the blood of a CTCL patient. Blood cells from the CTCL patient presented in Figure 3 were stained using monoclonal antibodies specific for different V-beta chains. The data indicate that in this patient, the tumour expressed V-beta-2.

Since it appears that immunoediting of T-cells can lead in the EL4 mouse model to immune escape, we aimed at analysing the stability of TCR expression in human lymphomas over time and through courses of immunomodulation therapy. In contrast to the mouse EL4 cell line, human T-cell lymphomas are difficult to maintain in an in vitro culture: malignant T cells undergo spontaneous apoptosis during culture and/or are outgrown by their benign T cell counterparts [32,33]. B-cell lymphomas require the B-cell receptor to survive, and it is postulated that the TCR is similarly required for the proliferation of T-cell lymphomas. Indeed, investigating clinical samples, we found that all cutaneous T-cell lymphomas express a TCR, and monoclonal rearrangement (Figure A3) of the TCR is specific for the diagnosis in 76.4–83.7% of cases and useful for the prognosis of the disease [34,35]. In addition, in most cases, the specific monoclonal rearrangement of the TCR remains unchanged during disease progression, remission and relapses, independent of the treatment modality used. As exemplified in Figure 3, the tumour cells in the blood of a patient responding to immunomodulation therapy by interferon-alpha (compare time point #2 to time point #1) and relapsing while off-therapy (compared time point #3 to time point #2) expressed the same clonotypic TCR over the course of the disease. No variation in expression could be detected over the one-year course of the fluctuating disease. Altogether, these results indicate that the TCR is an essential, stable and nonredundant structure for malignant T-cells. As a correlate, it can be speculated that vaccinating against the TCR in patients will not lead to immune escape.

## 4. Discussion

Our work demonstrates that a recombinant mRNA coding for the TCR CDR3 alpha and beta regions formulated in a vaccine liposome can provide some protection against a tumour cell line expressing the corresponding TCR. However, we also found that EL4 cells can downregulate the TCR and thereby escape anti-TCR immunity. EL4 cells grow in vitro very fast and without any addition of a stimulus to trigger the TCR. Thus, the TCR is probably not a driving mutation in this cell line. This feature dims the EL4 cell line as a relevant model for anti-TCR vaccine. In a study by Gonthier et al. [36], a peptide vaccine was injected in mice to trigger T-cells against a TCR V beta 12 variable region (not against a clonotypic CDR3 region). This vaccine protected 40 to 60% of the mice in a challenge with the V beta 12 expressing L12R4 T lymphoma cells. The reason why the vaccine failed to protect 40 to 60% of mice was not investigated. In the light of our present data, it could however be postulated that similarly to the EL4 cell, the L12R4 cells can lose expression of V beta 12 and therefore escape an immune response directed against this antigen. Still, both the present study and the study by Gonthier et al. indicate that vaccinating against a TCR can be used to provide some control of lymphoma in mice. In addition, we report in a patient that the TCR expression is stable over time and despite treatments. That suggests that as opposed to the EL4 situation, in CTCL, the TCR is a driver mutation and cannot be lost. Therefore, the strategy of the mRNA-based vaccine described in the present paper is expected to be a safe, specific and effective immunotherapy worth exploring for the treatment of patients with T-cell lymphomas. Our results warrant an investigation of the same anti-CDR3 mRNA vaccination design and experimentation in humanised mouse models that could be challenged with autologous T-cell lymphomas. These experiments are beyond the scope of this present study. Of note, although injections of mice are performed with 20 micrograms of RNA per injection, this formulation was previously demonstrated by Kranz et al. to be efficacious at a similar dose (7.2 to 29 micrograms) per injection in humans [12]. This type of vaccination can be advantageously combined with immune checkpoint inhibitors currently under evaluation in T-lymphomas [37,38,39,40] or established immunomodulatory approaches, especially interferon-alpha [41,42]. The possibility of vaccinating against the TCR to treat CTCL is further supported by other reports: Berger et al. [43], Zheng et al. [44] and Winter et al. [45] have shown that MHC class I epitopes derived from TCR chains expressed in human CTCL can be recognised by cytotoxic T-cells. Besides the intra-venous injection of liposomes bringing the unmodified (immunostimulating) mRNA to lymphoid organs as disclosed in mice and humans by Kranz et al. [12] and used here, the COVID-19 pandemic has seen the emergency approval of two formulations of modified (PseudoUridine: immunosilent mRNA) mRNA for intramuscular injections [11,46]. They have been shown to induce high antibody titres and activate T-cells [47]. However, one advantage of the intravenous injection of unmodified mRNA is the induction of type I interferon, which on its own has some anti-cancer effects and aids the penetration of T-cells into tissues [48]. Thus, so far, for cancer therapy, intra-venous injection of unmodified mRNA vaccines may be preferable as it bestows a dual immunotherapeutic effect against tumours: systemic type I interferon and potent adaptive T-cell immune responses. To conclude, our studies pave the way for a combination of immunomodulatory therapies and specific anti-TCR CDR3 mRNA-based vaccination for synergistic therapeutic immune control of T-cell lymphomas.

## Figures and Tables

**Figure A4:**
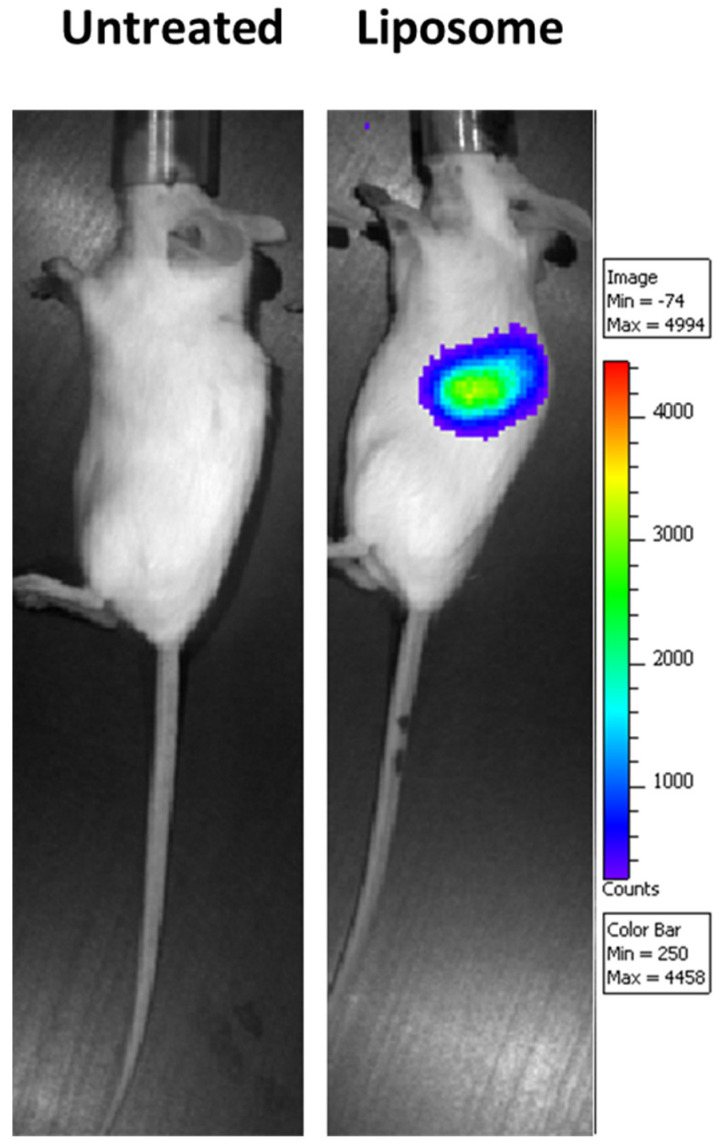
In vivo expression of mRNA. Synthetic mRNA coding firefly luciferase was formulated in the liposome as described in Materials and Methods. A mouse “Liposome” was injected intravenously with 10 µg of this formulated Luciferase-coding mRNA. Three hours after mRNA injection, in vivo bioluminescence imaging was performed on an IVIS Lumina instrument (PerkinElmer). Before each measurement, d-luciferin (Synchem) dissolved in PBS (15 mg/mL stock) and sterile filtered was injected (150 μg/g intraperitoneally) in both “Liposome” and untreated mice. Emitted photons from live animals were quantified 10 min post luciferin injections, with an exposure time of 3 min. The data show that the liposomal formulation used in this study efficiently delivers synthetic mRNA to the spleen.

## Data Availability

Data is contained within the article.
